# Monoacylglycerol Lipase: A Novel Potential Therapeutic Target and Prognostic Indicator for Hepatocellular Carcinoma

**DOI:** 10.1038/srep35784

**Published:** 2016-10-21

**Authors:** Junyong Zhang, Zuojin Liu, Zhengrong Lian, Rui Liao, Yi Chen, Yi Qin, Jinlong Wang, Qing Jiang, Xiaobo Wang, Jianping Gong

**Affiliations:** 1 The Second Affiliated Hospital of Chongqing Medical University, department of Urinary Surgery, Chongqing City, 400010, China; 2 The Second Affiliated Hospital of Chongqing Medical University, department of Hepatobiliary Surgery, Chongqing City, 400010, China; 3 McMaster University, department of Clinical Epidemiology and Biostatistics and Population Health Research Institution, Hamilton, Ontario, L8S4L8, Canada; 4 The First Affiliated Hospital of Chongqing Medical University, department of Hepatobiliary Surgery, Chongqing City, 400016, China; 5 Fuling Center Hospital, department of Emergency medicine, Fuling District, Chongqing, 408000, China; 6 Fuling Center Hospital, department of Hepatobiliary Surgery, Fuling District, Chongqing, 408000, China

## Abstract

Monoacylglycerol lipase (MAGL) is a key enzyme in lipid metabolism that is demonstrated to be involved in tumor progression through both energy supply of fatty acid (FA) oxidation and enhancing cancer cell malignance. The aim of this study was to investigate whether MAGL could be a potential therapeutic target and prognostic indicator for hepatocellular carcinoma (HCC). To evaluate the relationship between MAGL levels and clinical characteristics, a tissue microarray (TMA) of 353 human HCC samples was performed. MAGL levels in HCC samples were closely linked to the degree of malignancy and patient prognosis. RNA interference, specific pharmacological inhibitor JZL-184 and gene knock-in of MAGL were utilized to investigate the effects of MAGL on HCC cell proliferation, apoptosis, and invasion. MAGL played important roles in both proliferation and invasion of HCC cells through mechanisms that involved prostaglandin E2 (PGE2) and lysophosphatidic acid (LPA). JZL-184 administration significantly inhibited tumor growth in mice. Furthermore, we confirmed that promoter methylation of large tumor suppressor kinase 1 (LATS1) resulted in dysfunction of the Hippo signal pathway, which induced overexpression of MAGL in HCC. These results indicate that MAGL could be a potentially novel therapeutic target and prognostic indicator for HCC.

Metabolic reprogramming and proliferation are closely linked to tumorigenesis and progression^1^. Disorders associated with abnormal lipid metabolism are global health problems and have been found to be involved in a variety of diseases include hepatocellular carcinoma (HCC). HCC is the second leading cause of cancer-related deaths worldwide, with global incidences on the rise^2^,^3^. After the accidental discovery of the beneficial effects of statins on HCC^4^, lipid metabolism in the liver became an object of interest and scientists began to explore the relationship of lipid metabolism and tumor initiation and progression.

Liver cells obtain energy through fatty acid (FA) β-oxidation. However, according to Warburg’s theory, tumor cells favor glycolysis for energy production^5^. Identifying the altered energy supply mechanisms in HCC may reveal insights into the tumorigenesis and progression of this disease. As the producer of FFA, monoacylglycerol lipase (MAGL) is known for its role in the metabolism of endocannabinoid^6^. MAGL is also a key enzyme in lipid metabolism and participates in the last step of neutral lipid decomposition which resolves monoacylglycerol into fatty acid and glycerol. Furthermore, the MAGL-free fatty acid (FFA) pathway has recently emerged as a critical pathway that promotes tumor growth and invasion^7^.

MAGL is a key metabolism enzyme which regulates the network of FFAs in numerous aggressive tumors, such as colorectal cancer, neuroblastoma and nasopharyngeal carcinoma, by enabling tumor cells to mobilize and utilize FA from stored neutral fats^8^,^9^,^10^. Some of the released FFAs, including lysophosphatidic acid (LPA)^11^ and prostaglandin E2 (PGE2)^12^, were found to be involved in signal cascades which induce carcinogenesis and tumor progression. Considering the abundance and importance of FFAs and lipid metabolism in the liver, we predict that MAGL will be essential to the initiation and progression of HCC, perhaps more so than the other aforementioned cancers. Nevertheless, the role and mechanism of MAGL in HCC carcinogenesis and progression remain unclear.

In this study, we investigated the relationship between MAGL levels and clinical features of HCC patients. We also explored the mechanism of MAGL in HCC cell proliferation, invasion and apoptosis. Additionally, the reason for MAGL overexpression in HCC was investigated and the efficacy of targeted inhibition of MAGL *in vivo* was investigated to evaluate its potential value for HCC Therapy.

## Results

### MAGL levels are significantly higher in HCC and poorly differentiated in clinical samples

**Figure 1:**
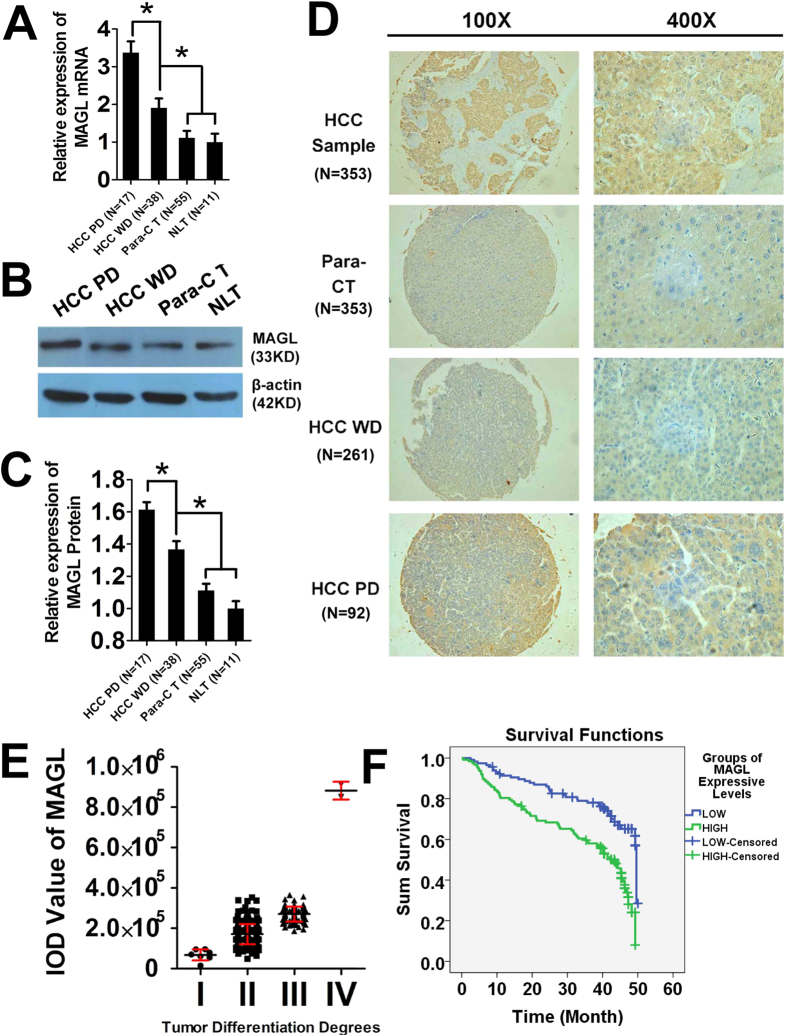
MAGL levels in tissue specimens and clinical relevance. MAGL mRNA levels (**A**) and protein levels (**B**,**C**) were examined by RT-PCR and western blot. Both mRNA levels and protein levels increased as the malignancy increased [from normal liver tissue (NLT) and para-carcinoma tissue (Para-CT) to HCC tissue well-differentiated (HCC WD) and HCC tissue poor differentiated (HCC PD)] in tissue specimens from HCC patients (*p < 0.05). These results were confirmed with IHC of TMA (**D**, 100× magnification; 400× magnification). MAGL protein was stained brown by IHC in the tissue slices, and stain was deeper when malignance increased in liver and tumor tissues. Patients were distributed according to MAGL protein level measured by IOD value by Image-Pro Plus 6.0 software (**E**). MAGL protein levels increased significantly with decreasing of tumor differentiation degrees^15^ of HCC (comparison among groups, p < 0.05). Kaplan Meier method for survival analysis (**F**) also demonstrated the MAGL low-expression group (MAGL IOD value <200000) showed significantly better survival than the MAGL high-expression group (MAGL IOD value >200000, p < 0.05).

In clinical patient samples (Fig. 1A–C), RT-PCR, Western Blots and IHC were used to evaluate MAGL mRNA and protein levels, respectively, along with TMAs (Fig. 1D). Significantly higher MAGL mRNA and protein levels were found in HCC samples when compared with non-HCC tissues (para-carcinoma tissue and normal liver tissue, *p < 0.05). This result was repeated in poorly differentiated HCC tissue compared with well differentiated HCC tissue (*p < 0.05). However, no significant difference of MAGL mRNA or protein levels was found between para-carcinoma and normal liver tissues.

### MAGL deteriorates the prognosis of HCC patients

TMA and IHC results indicated that MAGL levels were increased significantly with decreased degree of tumor differentiation in HCC [MAGL IOD values: I = 67758 ± 27697, II = 171019 ± 49765, III = 443878 ± 132285, IV = 848382 ± 91689 and NLT = 28024 ± 18200 (not showed), Fig. 1E]. This indicates that MAGL expression may be used to predict the degree of malignancy in HCC, confirming our previous findings^15^.

To further verify our results using TMA and IHC, logistic regression between MAGL protein levels and degrees of tumor differentiation, follow-up data of TMA, and Kaplan Meier method for survival analysis were completed. MAGL protein levels (MAGL IOD value), A-fetoprotein (AFP), tumor, node and metastasis (TNM) stage were significantly correlated to the degrees of tumor differentiation (p < 0.05, Table 1). Survival analysis also demonstrated MAGL low-expression group (MAGL IOD value < 200000) showed significantly better survival than MAGL high-expression group (MAGL IOD value > 200000, p < 0.05, Fig. 1F). Thus, MAGL protein levels could be considered as a biomarker for predicting tumor differentiation degree and prognosis.

Multiple linear regressions between MAGL protein levels, AFP, tumor sizes, tumor numbers, tumor embolus and survival times of HCC patients were performed using the following equation:


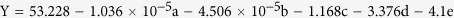


(a: MAGL IOD values, p = 0.010; b: AFP values, p < 0.001; c: tumor sizes, p < 0.001; d: tumor numbers, p = 0.038; e: tumor embolus or not, yes: 2, no: 1, p < 0.001.)

R^2^ = 0.166; Durbin-Watson: 1.989.

This equation showed a significant negative correlation between MAGL protein levels and other varieties.To further investigate the correlation between MAGL protein levels and survival of HCC patients, binary logistic regression of MAGL IOD values, one-year, and three-year survival of HCC patients were performed according to the follow-up data of the TMA. A significant direct correlation was found between MAGL IOD values and one-year survival of HCC patients (p = 0.010, overall percentage 88.4) but not three-year survival (p = 0.910, overall percentage 83.6) indicating that MAGL protein levels negatively affect short-term survival of HCC patients.

COX regression of MAGL protein levels and mortality risk factor of HCC were applied to evaluate the mortality risk of MAGL protein level to HCC patients (Table 2). Statistical results indicated that MAGL protein levels were associated with shorter survival time of HCC patients (p = 0.004).

### Downregulation of MAGL by small interference RNAs and pharmacological regulation

**Figure 2:**
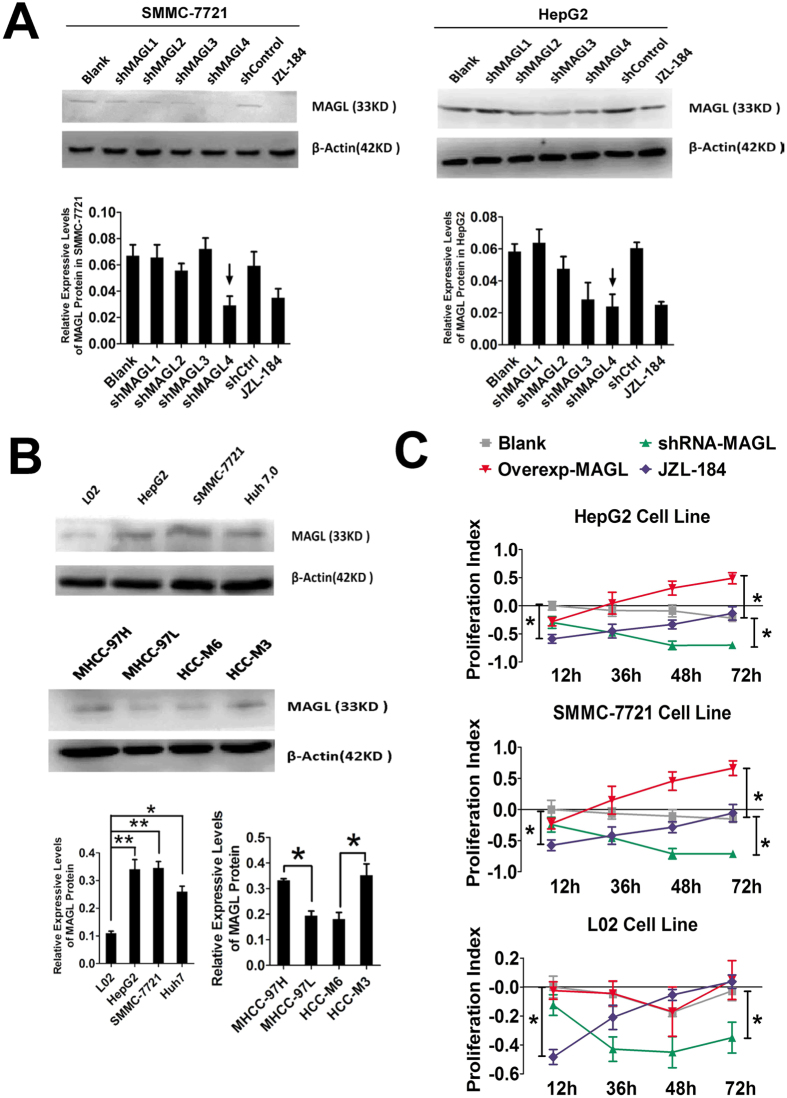
MAGL levels indicated the invasive ability and promoted proliferation of HCC cell lines. Western blot was utilized to evaluate the effects of MAGL protein knock-down of MAGL-shRNAs (shMAGLs), and specific inhibitor JZL-184 was used for positive control (**A**). ShMAGL4 was utilized as it exerted the strongest suppression of MAGL protein expression in both SMMC-7721 and HepG2 cell lines (indicated by small black arrows). MAGL levels also implied the malignance and invasive ability of HCC cell lines (**B**): MAGL protein levels were much higher in high invasive tumor cell lines (SMMC-7721, MHCC-97H and HCC-M3) than in low invasive cell lines (huh 7.0, MHCC-97L and HCC-M6, *p < 0.05) and normal liver cell line (L02, **p < 0.01). CCK8 assay was used to examine the proliferation ability changes when MAGL was regulated by iRNA, gene knock-in and JZL-184. MAGL was quickly inhibited by JZL-184 (blue lines; compared with blank group, black lines, *p < 0.05) which suppressed cell proliferation; gradually increasing, following drug metabolism. However, MAGL iRNA showed long-term and stable proliferation inhibition by MAGL knock-down (green lines; compared with blank group, *p < 0.05). In contrast, MAGL gene knock-in by MAGL gene-loading plasmid (pMAGL) transfection promoted proliferation of cell lines (red lines, compared with blank group, *p < 0.05).

HepG2, SMMC-7721 and Huh 7.0 were used to evaluate and compare the MAGL-knock down effect of MAGL-shRNA sequences. A specific inhibitor of MAGL, JZL184 (0.5 μg/μl, 24 h), was used as a positive control. Western blot analysis demonstrated that shRNA-4-MAGL and JZL-184 significantly downregulated MAGL protein expression (Fig. 2A), thus shRNA-4-MAGL was selected for further experiments.

### MAGL protein level is positively correlated with degree of malignancy in HCC cells

Western blot analysis of MAGL protein levels in various liver cell lines indicated that MAGL protein levels were significantly lower in L02 cells than HCC cell lines (Fig. 2B). MHCC-97H/L and HCC-M3/M6 represented high/low invasive HCC cell lines, respectively^16^. MAGL protein levels were significantly higher in HCC cell lines with high invasive scores, including MHCC-97H and HCC-M3 (Fig. 2B), compared to HCC cell lines with low invasive scores, including MHCC-97L and M6.

### MAGL promotes proliferation, invasion and apoptosis inhibition of HCC cell lines

ShRNA-MAGL transfected cell lines displayed significantly reduced IOD values after 48 hours, indicating that at the peak of shRNA transcription, there was a significant inhibition of HCC cell proliferation. This effect was also observed in the JZL184 group at 12 hours after treatment. Due to its quick and transient inhibition of MAGL, proliferation recovered gradually. Accordingly, MAGL knock-in plasmids induced MAGL-overexpression which significantly increased the proliferation of HCC cell lines but not L02 cells (Fig. 2C).

**Figure 3:**
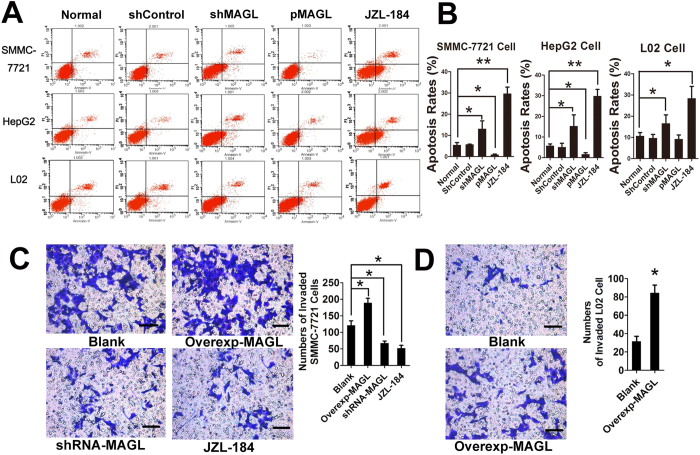
MAGL involved in anti-apoptosis of HCC cell lines and malignant change of liver cell. By flow cytometry (**A**), in all cell lines, inhibition MAGL protein expressions and function by iRNA or JZL-184 significantly elevated cell apoptosis rates (**A**,**B**, *p < 0.05, **p < 0.01). Correspondingly, increasing protein levels of MAGL by gene knock-in reduced apoptosis rates of HCC cells except in normal liver cells (**B**, *p < 0.05). This outcome was also found in matrigel invasion assay. HCC cell line SMMC-7721 showed enhanced invasion ability when overexpressed MAGL by gene knock-in, and impaired invasion ability when MAGL was suppressed by iRNA or JZL-184 (**C**), *p < 0.05). Interestingly, a certain level of invasion ability was observed in normal liver cell line L02, which was unable to invade after overexpressed MAGL by gene knock-in (**D**, *p < 0.05). This result implied MAGL is involved in malignant change or tumorigenesis.

Flow cytometric analysis of apoptosis indicated MAGL inhibition, though both shRNA and JZL184 treatment, significantly increased apoptosis of HCC cell lines and normal liver cell line. Overexpression of MAGL inhibited cell apoptosis of HCC cell lines but not normal liver cell line (Fig. 3A,B). Taken together these results indicate that MAGL expression promotes proliferation and inhibits apoptosis in HCC cells.

Due to the weaker invasive abilities of HepG2 and Huh 7.0 cell lines, SMMC-7721 was used for matrigel invasion assay. When MAGL was downregulated by shRNA or JZL184 in SMMC-7721 cells, a significant decrease in cell invasion was observed, indicating that downregulation of MAGL impaired invasive capacity of SMMC-7721 cells. In agreement with these results, overexpression of MAGL promoted invasion of SMMC-7721 through the matrigel (Fig. 3C). These results demonstrated that MAGL enhances the invasive capacity of HCC cells. Interestingly, although overexpression of MAGL did not promote proliferation of normal liver cell line L02, significant invasion ability was found in L02 cells that overexpressed MAGL (Fig. 3D). The result implied that MAGL may be involved in initiation and progression of HCC.

### Nude mouse tumorigenicity assay

**Figure 4:**
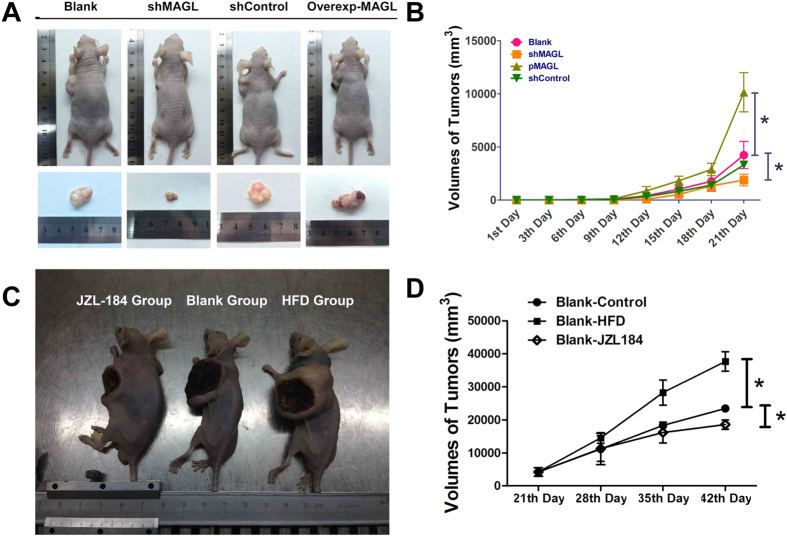
Tumorigenesis assay and curative effect test of JZL-184 in Nude mice. Subcutaneously implanted HCC model in nude mice was established with SMMC-7721 cells which stably overexpressed MAGL protein by pMAGL transfection or knocked-down MAGL by shMAGL. After feeding routinely for 21 days, HCC nude mice were established (**A**, gross samples). Largest tumors were found in nude mice which were injected subcutaneously with MAGL-overexpressed SMMC-7721 cells (**A**,**B**, breen line, *p < 0.05). Knock-down MAGL remarkably inhibited tumor growth which showed the smallest tumor sizes (**A**,**B**, orange line, *p < 0.05). Subcutaneously implanted HCC model in nude mice with wide-type SMMC-7721 cells were fed with a high fat diet (HFD) or JZL-184 oral administration for 21 days. Gross samples were showed in picture (**C**), from left to right: JZL-184 oral administration group, blank group and HFD group. HCC bearing nude mice fed with HFD showed a significant increase of tumor sizes (**D**, line with squares, *p < 0.05). Meanwhile, HCC animal models with JZL-184 oral administration displayed impaired tumor growth ability (**D**, line with squares, *p < 0.05).

SMMC-7721 cells which stably expressed MAGL-shRNA (shMAGL group) or overexpressed MAGL protein (pMAGL group) were used in tumorigenicity assays in nude mice. After subcutaneous injection of cell suspension into nude mice, tumor volumes increased over time. Inhibition of MAGL slowed tumor growth, but overexpression of MAGL accelerated the growth of tumors. After 21 days, differences of tumor volumes between shMAGL group, pMAGL group, and blank group were significantly different (p < 0.05, Fig. 4A,B).

In the trial of MAGL-targeted pharmacological inhibition, JZL-184 oral administration significantly inhibited tumor growth of nude mice, while high fatty diet (HFD) promoted tumor growth (p < 0.05, Fig. 4D). After 42 days, gross samples of nude mice were collected and analyzed. The largest and smallest tumor sizes were recorded in HFD group and JZL-184 P.O. group, respectively (Fig. 4C, from left: JZL-184 P.O. group, blank and HFD group), indicating that MAGL promotes tumorigenicity of SMMC-7721 cells.

### Analysis of MAGL-PGE2/LPA pathway in HCC cells

**Figure 5:**
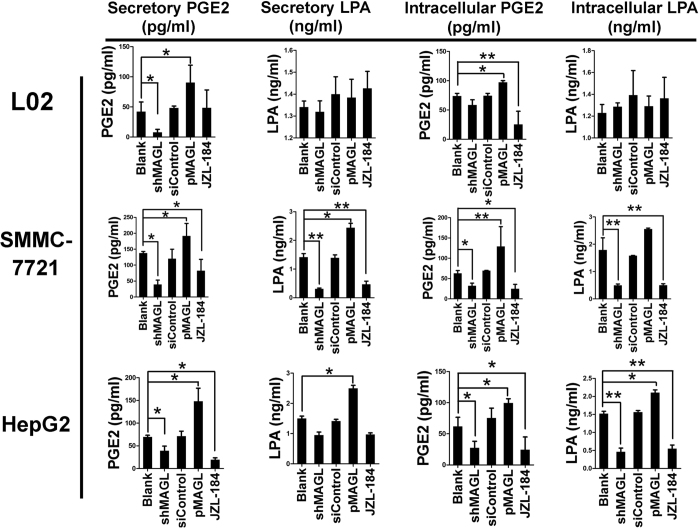
MAGL promotes HCC cell proliferation and invasion by MAGL-PGE2/LPA pathway. Secretive and intracellular levels of both PGE2 and LPA approximately showed the same trend of the expression levels and function of MAGL after our moderation in HCC cell lines. However, only PGE2 showed correlation with MAGL in liver cell line L02. When MAGL was knocked-down by shRNA or inhibited by JZL-184, PGE2 secretory levels and intracellular levels in both HepG2 and SMMC-7721 cell lines, LPA intracellular levels in both HCC cell lines, and secretory level of LPA in SMMC-7721 significantly decreased. Secretory level of LPA in HepG2 did not show the same result. Knock-in MAGL these cell lines elevated both PGE2 and LPA levels regardless of cell line types, but LPA levels in L02.

ELISAs were performed to detect the levels of two potential downstream effector molecules of MAGL, PGE2 and LPA, in both secreted and intracellular levels. A similar trend in the secreted and intracellular levels of PGE2 and LPA as MAGL was observed in HCC cell lines when MAGL levels were modulated. However, only PGE2 levels correlated with MAGL in the normal L02 liver cell line (Fig. 5). Upon knockdown or pharmacologic inhibition of MAGL, secretory and intracellular levels of PGE2 decreased in both HepG2 and SMMC-7721 cell lines. However, only intracellular levels of LPA were reduced in both HCC cell lines. Secretory level of LPA was only significantly decreased in SMMC-7721 but not HepG2 cells. Conversely, MAGL upregulation in all cell lines led to significant increases in both PGE2 and LPA levels in all HCC and normal liver cells, except for LPA levels in L02 cells. These results demonstrate that tissue-specific differences existed in the levels of both PGE2 and LPA and these differences were controlled by MAGL expression. These results support the notion that both PGE2 and LPA are downstream products of MAGL.

### Promoter of LATS1 methylation induces MAGL overexpression by blocking Hippo signal pathway

**Figure 6:**
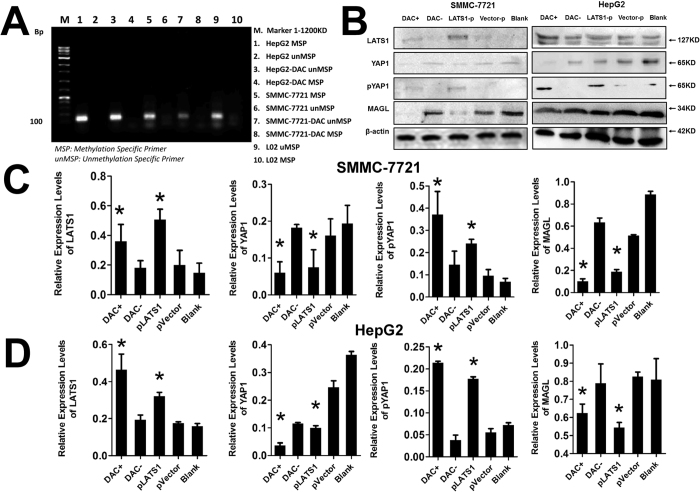
Promoter of LATS1 methylation induced MAGL overexpression by blocking the Hippo signal pathway. Methylation special primers were detected with MSP in all HCC cell lines but in L02 by MSP. Unmethylation special primers were only detected in L02. After treatment by DAC, methylation special primers decreased in HCC cell lines while unmethylation special primers increased (**A**). In HepG2 and SMMC-7721 cells (**B**–**D**), expression of LATS1 protein was suppressed. Demethylation of LATS1 promoter by DAC and knock-in LATS1 gene by plasmid transfection elevated LATS1 protein levels, which confirmed our findings that methylation of LATS1 promoter induced lost-expression of LATS1 protein in HCC. In HepG2 and SMMC-7721 cell lines, regulation of LATS1 with these methods induced significant changes in downstream signals YAP protein levels in a negatively correlative manner and pYAP protein levels in a positively correlative manner. Finally, due to methylation of LATS1 promoter and lost expression of LATS1, YAP protein could not be phosphorylated and detained in cytoplasm effectively. Excess YAP imported into nucleus and induced overexpression of MAGL.

MSP was utilized to evaluate the methylation of the LATS1 promoter in HCC cell lines HepG2 and SMMC-7721 and normal liver cell line L02 (Fig. 6A). Methylation specific primers were detected in all HCC cell lines but in L02 liver cells. Non-methylated specific primers, however, were only detected in the L02 normal liver cell line. To further confirm the methylation patterns of the LATS1 promoter in HCC cell lines, DAC was used to demethylate the LATS1 promoter in HCC cell lines. DAC treatment of HCC cells led to a decrease in methylation specific primers while non-methylation specific primers increased. In summary, methylation of LATS1 promoter was observed in HCC cells but not in normal liver cells and DAC led to demethylation of LATS1 in HCC cells.

**Figure 7:**
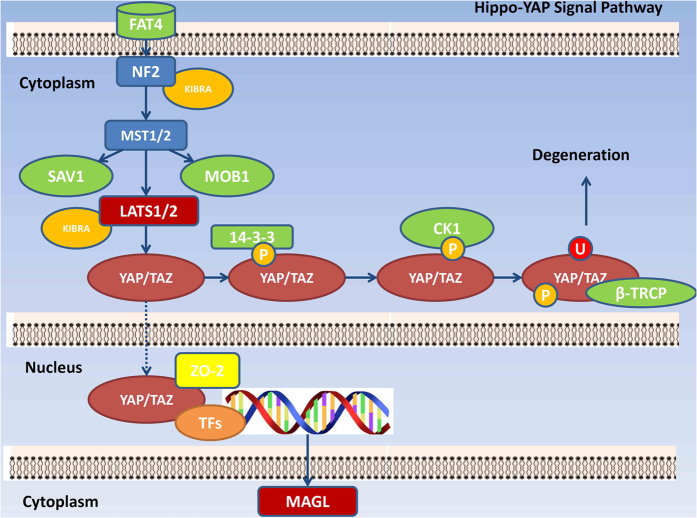
MAGL in Hippo-YAP signal pathway.

In HCC cell lines HepG2 and SMMC-7721 (Fig. 6B–D), expression of LATS1 protein was suppressed. Demethylation of LATS1 promoter by DAC and Knock-in LATS1 gene by plasmid transfection elevated LATS1 protein levels, which confirmed our findings that methylation of the LATS1 promoter in HCC cells induced decreased expression of the LATS1 protein. Deregulation of LATS1 in HepG2 and SMMC-7721 cell lines using the methods described above led to significant changes in the YAP and pYAP protein levels. LATS1 and YAP displayed a negative correlation, whereas, LATS and pYAP demonstrated a positive correlation. These results demonstrated LATS1 is involved in the phosphorylation of YAP which induces nucleus retention of YAP and advancement of the Hippo-YAP signaling pathway^17^. These results agree with the report by Tang *et al.*^18^ which demonstrated that YAP induced MAGL mRNA transcription. In conclusion, in HCC cell lines, methylation of the LATS1 promoter induced downregulation of LATS1 and a subsequent decrease in the phosphorylation and nuclear entry of YAP which may induce excessive transcription of MAGL (Fig. 7).

## Discussion

Since Nomura *et al.* first reported the tumor promoting effects of MAGL^7^, increasing studies investigating the relationship between MAGL, carcinogenesis, and tumor progression have been performed to reveal insights into the relevant mechanisms. Until now, the role of MAGL in tumorigenesis and progression remains controversial due to the fact that tumor suppressing effects of MAGL are observed in some colorectal cancers^8^,^19^. In prostate cancer, ovarian cancer and colorectal cancer MAGL has been shown to be a key enzyme in the lipid metabolism network, promotes tumor progression by supplying FFA for β-oxidation, provides components to build cell structures and effector molecules which are involved in cell proliferation, invasion, apoptosis resistance and stemness. Additionally, MAGL facilitates tumor growth by degrading 2-arachidonoylglycerol (2-AG) and inhibiting activation of cannabinoid receptor-1 (CB1)^20^. In contrast, the anti-tumor properties of MAGL have been shown to be mediated by the PI3K-ATK signal pathway which suppresses anchorage-independent growth (AIG) and metastasis in certain tumors^18^,^19^.

As a central organ in lipid metabolism, the liver is likely to be affected by aberrant lipid metabolism. Furthermore, disorders of lipid metabolism demonstrate a significant correlation with HCC^21^,^22^,^23^. Therefore, due to the pivotal role of MAGL in lipid metabolism, its likely involvement in carcinogenesis and progression of HCC were investigated. This study, for the first time, demonstrated that MAGL expression levels were positively correlated with aggressiveness of HCC cell lines and negatively correlated with HCC tissue differentiation degrees. Additionally, MAGL was shown to play an important role in proliferation, apoptosis inhibition, invasion, and tumorigenesis of HCC cells. The tumor-promoting functions of MAGL were shown to be caused by the downstream effector molecules, LPA and PGE2. Furthermore, we demonstrated that the Hippo signaling pathway was responsible for MAGL overexpression in HCC cells. Specifically, methylation of the LATS1 promoter induced LATS1 protein downregulation, and subsequently, decreased phosphorylation and increased nuclear entry of YAP, which induces transcription of MAGL.

Inhibition of MAGL expression and function with shRNA or pharmacological methods, respectively, suppressed HCC growth and progression *in vivo*. Thus, we propose that MAGL may be a novel HCC therapeutic target due to its numerous significant tumor promoting effects. Furthermore, we found that MAGL could be considered as a prognostic indicator for HCC since increased MAGL levels correlate with decreased HCC tissue differentiation degree.

To go a step further, an equation was designed with multiple linear regressions between MAGL protein levels, AFP, tumor sizes, tumor numbers, and tumor emboli to roughly calculate the survival time of HCC patients. Due to the use of data from follow-up times that spanned only 48 months, the R^2^ value of this equation was small and the linear correlation is not strong, however, with larger samples and longer study times we do believe a stronger correlation will be reached. We demonstrated that an increase of MAGL protein levels was correlated with decreased short-term survival (one-year survival) of HCC patients in binary logistic regression. The shortened survival time of HCC patients associated with MAGL was confirmed with COX regression, and may be considered as an independent mortality risk of HCC patients. Taken together, these data support the use of MAGL as a prognostic indicator for HCC patients along with a potential therapeutic target.

Despite the recent research indicating the relationship between MAGL and tumor progression, the mechanisms of the protumor activity of MAGL are unknown. Here, we provide insights regarding the mechanism of action of MAGL in HCC, however, some key questions remain unanswered. Are there other mechanisms, aside from the MAGL-LPA/PGE2 and MAGL-2-AG-CB1 pathways, that are involved in the protumor effects of MAGL? Aside from tumor cell proliferation and invasion, what effect does MAGL have on tumor development and progress, for example, does it modulate tumor cell metabolism through the MAGL-FFA pathway? Finally, will MAGL be an effective therapeutic target and prognostic indicator for HCC? It remains unclear whether histological examination may be suitable for clinical application of HCC diagnosis and prognosis prediction but it may be worth investigating whether serum levels of MAGL may provide a more reliable and convenient clinical test for HCC diagnosis and prognosis.

## Methods

### Patients and tissue microarrays

Tissue microarray of 353 HCC patients was gifted from the Affiliated Zhongshan Hospital of Fudan University with complete patients ‘authorization and full ethical approval of the Institutional Clinical Ethics Review Board of Fudan University (Table 3). Fifty pairs of HCC and para-carcinoma tissue (untreated, 36 males and 14 females, aged from 35–84), 11 normal liver tissue (traumatic hepatorrhexis) samples were obtained from the Second Affiliated Hospital of Chongqing Medical University and Fuling Center Hospital with histopathological confirmation, prior patient consent, and the approval of the Institutional Clinical Ethics Review Board in the Second Affiliated Hospital of Chongqing Medical University. All experiments with human tissue samples mentioned above were carried out in accordance with the consent and approval aforementioned, and the informed consent was obtained from all subjects. None of the patients received preoperative chemotherapy or radiation therapy. We defined tumor differentiation degree I and II as HCC well-differentiated (HCC WD), III and IV as HCC poor-differentiated (HCC PD)^15^.

### Immunohistochemistry analysis

Sample collection and immunohistochemistry (IHC) assays were performed according to previously described conventional methods (reference: Rego, S. L. *et al.* Methods. 2015). Tissues were fixed, processed, embedded, sectioned on microscope slides, and blocked. Slides were incubated at 4 °C overnight with polyclonal MAGL antibodies in dilution buffer (1:200; Abcam, USA). The avidin–biotin technique was performed according to manufacturer’s recommendations with secondary biotinylated antibody and avidin-peroxidase complex kit (Boster, China).

### Cell culture

The HCC cell lines, SMMC-7721, HepG2, Huh 7.0 (from Institute of Life Science, Chongqing Medical University), MHCC-97L, MHCC-97H, M3 and M6 (from the Affiliated Zhongshan Hospital of Fudan University) were cultured in Dulbecco’s Modified Eagle Medium (DMEM) high glucose (Hyclone, USA) supplemented with 10% fetal bovine serum (Hyclone, USA). Liver cell line L02 (from Institute of Life Science, Chongqing Medical University) was cultured in Roswell Park Memorial Institute 1640 (RPMI-1640) medium (Hyclone, USA). All cells were maintained in a humidified 37 °C incubator with 5% CO_2_. SMMC-7721, HepG2 and Huh 7.0 HCC cell lines were cultured using conventional methods and MHCC-97L/H and M3/M6 cell lines were paired HCC cell lines with low/high invasive abilities^16^.

### RNA interfering and transfection

Four pairs of small hairpin ribonucleic acid (shRNA) directed against different encoding region of MAGL transcript were designed and synthesized (Invitrogen, USA). Nucleotide sequences are shown in Table 4. Plasmids carrying MAGL or LATS1 gene codes and blank plasmid vector (Genepharma, China) were used as controls. Transfection was performed on cells in 24-well plates. Briefly, cells were transfected with 2 μl (1 μg/μl) shRNA per well using our self-developed transfection agent Large-scale mesoporous silica nanospheres (LPMSNs)^13^. Transfection was performed at least five times in each experiment and each experiment was repeated at least three times.

### Western Blot analysis

Cells were seeded in 6-well plate and treated with JZL184 (0.5 μg/μl, Sigma, USA) for 12 h, DAC (5-Aza-2′-deoxycytidine, 1 M, Sigma, USA) for 72 h or transfected with shRNA for 72 h, then harvested for total protein extraction and estimation. Polyacrylamide gel electrophoresis (PAGE) was performed and PAGE gels transferred to nitrocellulose membranes using previously described protocols (reference: Rego, S. L. Angiogenesis, 2014). After transfer membranes were blocked with 5% nonfat milk, incubated with polyclonal antibodies for MAGL, LATS1, YAP1 or pYAP1 (Abcam, USA) respectively, and a monoclonal antibody for β-actin (Boster, China) was used as a loading control. After incubation with horseradish peroxidase conjugated secondary antibody (Boster, China), membranes were developed using the enhanced chemiluminescence (ECL, Boster, China) detection system (Amersham, USA).

### RT-PCR and methylation specific PCR (MSP)

The integrity of total RNAs was test by agarose gel electrophoresis (AGE). Primers used to amplify fragments using real time-polymerase chain reaction (RT-PCR) are shown in Table 5. RT-PCR was performed using StepOnePlus™ RT-PCR System (Applied Biosystems, USA) with SYBR-Green PCR Master Mix (Applied Biosystems, USA) using routine methods^14^. MSP was performed utilizing standard methods. Data was analyzed with StepOne Software V2.1 (Applied Biosystems, USA).

### Cell proliferation analysis

Cell Counting Kit-8 (CCK8) method was used to analyze cell proliferation after modulating MAGL. Cells were plated in 96-well plates at a density of 10,000/well and cultured for 24 hours. Transfection or JZL-184 treatment was performed directly in 96-well plates. 10 μl CCK8 solution was added to each well at corresponding time points and plates incubated for one hour. The optical density was measured at 450 nm using a Spectrophotometer (Beckman, USA).

### Cell apoptosis analysis

Flow cytometry was utilized for cell apoptosis analysis. After treatments cells were digested and washed with phosphate buffered saline (PBS). 5 μl FITC-Annexin and 5 μl PI (250 μg/ml) were added into the cell suspensions, and incubated on ice in dark for 10 minutes. Next, cells were washed with PBS two times, and fluorescence analyzed using a flow cytometry instrument (BD Bioscience, USA).

### Enzyme-linked immunosorbent assay (ELISA)

In order to confirm the involvement of LPA and PGE2 in MAGL pathway, ELISAs were used to detect the levels of LPA and PGE2 in cell culture mediums (secreted levels) and cell lysate (intracellular levels). Cell culture media containing secreted proteins was collected 24 hour after treatments. Cell lysis buffers were prepared by repetitive freeze thaw method after transfection for 48 hours and JZL184 treatment for 12 hours. ELISA was operated according to manufacturer’s recommendations (Cusabio Biotech, China).

### Matrigel invasion assay

Matrigel was used to coat the 8.0 μm microporous transwell membranes. Cell suspensions (2.5 × 10^5^ per 100 μl) were re-suspended in serum-free DMEM and added into upper well of the transwell insert and 750 μl DMEM with 20% FBS was added to the lower well. Plates were then incubated at 37 °C, 5% CO_2_ for 24 hours. Next, membranes were fixed with 4% paraformaldehyde, washed with PBS two times and stained with 0.1% crystal violet for 30 minutes. Cells that invaded through transwells were counted using a bright field microscope.

### Animal model

Four week old male BALB/c node mice were purchased from Animal Center of Chongqing Medical University. All of the animal experimental protocols were approved and supervised by the Institutional Animal Care and Use of Committee at Chongqing Medical University and were carried out in accordance with the “Experimental Animal Management Approach of Chongqing City” (the 195^th^ Order of Chongqing Municipal Government, 2006) and “Experimental Animal Interim Provisions of Chongqing Medical University” (2005-2-CQMU-LAC). The mice were maintained in a pathogen-free facility with a 12-h light, 12-h dark cycle and were provided food (both normal diets and high fat diets) and water ad libitum. All efforts were made to minimize animal suffering, including euthanasia, and to reduce the number of animal used. In order to evaluate the tumorigenesis abilities of cells after MAGL manipulation, stably transfected SMMC-7721 (MAGL-knockdown or MAGL-overexpressed) were screened with G418 for nude mouse tumorigenicity assay. Cell suspension with 1 × 10^6^/ml was injected subcutaneously into subscapular region of nude mice. Tumor size was recorded every three days and calculated as ab^2^/2 (a stands for the long diameter, b stands for the short diameter). JZL-184 oral administration (50 mg/kg, qod) was used for of MAGL-targeted inhibition therapy *in vivo*.

### Statistical analysis

The data were analyzed using the Statistical Package for the Social Sciences 18.0 (SPSS, USA) with a p < 0.05 taken as statistically significant. The measurement data were expressed as mean ± SD of at least three experiments. Rank sum test and chi-square test were used for categorical data, and the one-way analysis of variance (ANOVA) test was used for continuous data. The power analysis for the final sample size was calculated with Graph Pad Stat Mate (Graph Pad Software, CA).

## Additional Information

**How to cite this article**: Zhang, J. *et al.* Monoacylglycerol Lipase: A Novel Potential Therapeutic Target and Prognostic Indicator for Hepatocellular Carcinoma. *Sci. Rep.* **6**, 35784; doi: 10.1038/srep35784 (2016).

## Figures and Tables

**Table 1:** Logistic regression of MAGL protein levels and degrees of tumor differentiation.

Effect	Model Fitting Criteria	Likelihood Ratio Tests		
	−2 Log Likelihood of Reduced Model	Chi-Square	df	Sig.
Intercept	296.001	68.217	4	0.000
**AFP**	**243**.**812**	**16**.**028**	**4**	**0.003**
Tumor embolus	234.274	6.490	4	0.165
**IOD Value**	**452**.**683**	**224**.**899**	**4**	**0**.**000**
Tumor Number	230.108	2.324	4	0.676
Tumor size	235.146	7.362	4	0.118
**TNM stage**	**242**.**9437**	**15**.**159**	**4**	**0.004**

The chi-square statistic indicating a difference in −2 log-likelihoods between the final model and a reduced model. The reduced model is formed by omitting an effect from the final model. The null hypothesis is that all parameters of that effect are 0.

**Table 2:** COX regression of MAGL protein levels and mortality risk factor.

Omnibus Tests of Model Coefficients									
−2 Log Likelihood	Overall (score)			Change From Previous Step			Change From Previous Block		
	Chi-square	df	Sig.	Chi-square	df	Sig.	Chi-square	df	Sig.
1241.504	8.582	1	0.003	5.900	1	0.015	5.900	1	0.015
Variables in the Equation									
	B	SE	Wald	df	Sig.	Exp(B)			
IOD Value	0.000	0.000	8.272	1	**0**.**004**	**1**.**000**			

**Table 3:** Characteristics of patients in TMA.

Patients	Nos
Gender	
Male	300
Female	53
Age	
<60	260
≥60	93
Tumor Sizes	
<5.0 cm	202
≥5.0 cm	151
<5.0 cm	202
Tumor Number	
Single	308
Multiple	45
Clinical Stages	
I	244
II	101
III	8
Tumor Embolus	
YES	259
NO	94
Differentiated degree15	
I	7
II	254
III	90
IV	2
AFP(μg/L)	
<400	213
≥400	140
Tumor-Free Survival(Month)	
<12	82
≥12, <24	48
≥24, <48	186
≥48	37
<12	82
Recurrence	
YES	164
NO	189

**Table 4:** The nucleotide sequences of shRNAs.

	Template sequences
ShMAGL-1	Sense 5′-CACCGGATGGTAGTGTCTGACTTCCTTCAAGAAGAGAGGAAGTCAGACACTACCATCCTTTTTTG-3′
	Antisense 5′-GATCCAAAAAAGGATGGTAGTGTCTGACTTCCTCTCTTGAAGGAAGTCAGACACTACCATCC-3′
ShMAGL-2	Sense 5′-CACCGCCAATCCTGAATCTGCAACATTCAAGAGATGTTGCAGATTCAGGATTGGCTTTTTTG-3′
	Antisense 5′-GATCCAAAAAAGCCAATCCTGAATCTGCAACATCTCTTGAATGTTGCAGATTCAAGGATTGGC-3′
ShMAGL-3	Sense 5′-CACCGCCTACCATGTTCTCCACAAGTTCAAGAGACTTGTGGAGAACATGGTAGGCTTTTTTG-3′
	Antisense 5′-GATCCAAAAAAGCCTACCATGTTCTCCACAAGTCTCTTGAACTTGTGGAGAACATGGTAGGC-3′
ShMAGL-4	Sense 5′-CACCGCATGTGGATTCCATGCAGAATTCAAGAGATTCTGCATGGAATCCACATGCTTTTTTG-3′
	Antisense 5′-GATCCAAAAAAGCATGTGGATTCCATGCAGAATCTCTTGAATTCTGCATGGAATCCACATGC-3′
Negative Control	Sense 5′-CACCGTTCTCCGAACGTGTCACGTTTCAAGAGAACGTGACACGTTCGGAGAATTTTTTG-3′
	Antisense 5′-GATCCAAAAAATTCTCCGAACGTGTCACGTTCTCTTGAAACGTGACACGTTCGGAGAAC-3′

**Table 5:** Amplified fragments of RT-PCR.

Gene	Primer sequences	Product size (bp)
MAGL	5′-TCTGACTTCCACGTTTTCGTC-3′	179 bp
	5′-AGAACCAGAGGCGAAATGAGT-3′	
β-actin	5′-CTGAGAGGGAAATCGTGCGT-3′	208 bp
	5′-CCACAGGATTCCATACCCAAGA-3′	
Methylation primers	5′-GGAGTTTCGTTTTGTC-3′	138 bp
	5′-CGACGTAATAACGAACGCCTA-3′	
Unmethylation primers	5′-TAGGTTGGAGTGTGGTGGT-3′	121 bp
	5′-CCCAACATAATAACAAACACCT-3′	
